# Digital analysis of the relationship between maximum bite force and 3-dimensional arrangement of mandibular first molars

**DOI:** 10.4317/jced.62037

**Published:** 2024-12-01

**Authors:** Anh Ho-Quynh Nguyen, Oanh Ngoc-Hoang Nguyen, Duy Le Nguyen, Tin Si Ho, Khai Dinh-Thien Pham, Khoa Dang Nguyen, Nam Cong-Nhat Huynh, Hung Trong Hoang

**Affiliations:** 1 Doctor, Lecturer. Faculty of Odonto-Stomatology, University of Medicine and Pharmacy at Ho Chi Minh City, 652 Nguyen Trai Street, Ward 11, District 5, 749000, Ho Chi Minh City, Vietnam; 2 Doctor. Faculty of Odonto-Stomatology, University of Medicine and Pharmacy at Ho Chi Minh City, 652 Nguyen Trai Street, Ward 11, District 5, 749000, Ho Chi Minh City, Vietnam

## Abstract

**Background:**

Bite force is one of the important factors that determine the chewing efficiency of molars. This study aimed to investigate the relationship of the maximum bite force (MBF) to the 3-dimensional (3D) arrangement of the first mandibular molars in Angle’s class I healthy adults using a digital protocol.

**Material and Methods:**

Subjects were 33 adults (16 males and 17 females) aged 18-25, with Angle’s class I occlusions and healthy dentitions. MBF was recorded by a digital occlusal force gauge (BFM 4th generation, Vietnam). 3D models were scanned using TRIOS 3 intra-oral scanner (3Shape, Denmark), and analyzed using Geomagic Design X software (Artec, Luxembourg). The digital measurement included two steps: reorientation and measuring. First, all the virtual upper models were reoriented into the same Oxyz coordinates using 3 landmarks: one at the incisive papilla and two at the intersection of the palatal sulci of the ﬁrst permanent molars with the gingival margin. Next, 3D position of the first mandibular molars was measured using crown angulation (CA), crown inclination (CI), and depth of curve of Spee (DCOS). t-tests were conducted to compare the mean values between sides and gender. Pearson’s correlation coefficient was performed to evaluate the statistical relationships.

**Results:**

Mean MBF was 619.66±36.25 N; mean DCOS was 1.73±0.30 mm; mean CA and CI were 2.21±1.70° and -29.65±6.93°, respectively. Male adults showed greater MBF than females significantly. Correlation coefficient between MBF and CA was -0.60, and between MBF and CI was -0.43 significantly. MBF and DCOS were not related.

**Conclusions:**

MBF was influenced by gender and the first mandibular molar CA and CI. Hence, it should be considered carefully when the treatment plan includes restoration or any change in the position of the first mandibular molars.

** Key words:**Bite force, curve of Spee, crown angulation, crown inclination, digital dentistry.

## Introduction

Maximum bite force (MBF) serves as an important indicator of the masticatory system’s functional state as it reflects the coordinated interaction among various components, including muscles, bones, and teeth. The masticatory system is a whole functional unit that includes: the dentition and periodontium, jaw bones and muscles, temporomandibular joints, and related structures, all of which play a vital role in humans’ physiological and social functions. As evaluating the integrity and function of the masticatory system is an important task of dental practitioners, numerous researchers have been interested in MBF (1) Considering the total force generated during mandibular movements as well as measuring it is useful in the diagnosis of masticatory system disorders. Besides, measuring the bite force at each tooth helps evaluate restorations, design implants, and choose the appropriate filling materials (2).

In addition to looking into each individual component, understanding the interactions between the components of the masticatory system helps practitioners develop more comprehensive treatment plans. A previous study indicated that the bite force might be affected by the arrangement of teeth and the relationship between the two jaws (3,4). In jaw-closing movement, with the presence of the curve of Spee (COS), teeth are in maximal intercuspation almost at the same time, with the same intensity and angle, assisting the teeth and periodontal tissues to bear appropriate forces when chewing. A study examined the dry skulls of primates and humans, suggesting that COS had an important contribution to the biomechanical function related to mastication (5). Also, crown angulation (CA) and inclination (CI) are two important factors in Andrews’s six keys to achieving normal occlusion. Thus, in orthodontic treatment, full-mouth rehabilitation or removable full dentures, teeth arrangement, and occlusal curves need to be taken into thorough consideration to acquire optimal chewing efficiency after treatment (5-7). The correlation between bite force and tooth direction as well as occlusal curves has been investigated in different populations but not yet in Vietnam. Besides, almost all previous studies used conventional measuring methods on plaster casts. With the development of technology, the parameters can be measured digitally with high accuracy and reliability (8). Therefore, we applied digital methods to conduct this study to investigate the MBF at first molars in healthy adults with Angle’s class I occlusion and assess its relationship with the three-dimensional (3D) arrangement of mandibular first molars.

## Material and Methods

-Participant recruitment

The present descriptive and analytic cross-sectional study was carried out from September 2021 to July 2022, with the approval of the Institutional Ethics Committee of the University [full name redacted] (Approval 462 on August 30th, 2021). The study consisted of 33 subjects: 16 males and 17 females; age 18-25, mean 22.03 years. Only those subjects who willingly participated in the study and provided written consent were included. All subjects had to meet the following inclusion criteria: full permanent dentition, including the second molars (at least 28 teeth) and bilateral Angle’s Class I first molar relation, average-face type. Exclusion criteria were anterior or posterior crossbite, a history of previous or current orthodontic treatment, maxillo-facial surgery, craniofacial trauma, endodontic treatment, restorations, or fillings on first molars, dental restorations cover cusps on mandibular canines or second molars, severe attrition, periodontal diseases, or temporomandibular disorders.

-Study procedure

**Figure 1:**
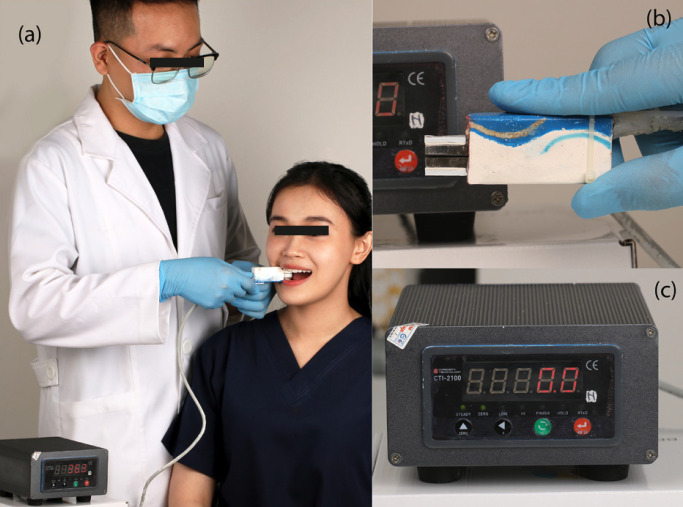
(a) Biteforce Meter BFM 4th generation, Vietnam, certificate of calibration number SG19-0390 by Sai Gon technology center for measurement and calibration; (b) Biteforce Meter BFM 4th generation biting transducer; (c) Biteforce Meter BFM 4th generation screen display.

All selected subjects followed the procedure: MBF at first molars was recorded by a digital occlusal force gauge (BFM 4th generation, Vietnam, certificate of calibration number SG19-0390 by Sai Gon technology center for measurement and calibration) (Fig. 1). 3D virtual models of each participant were acquired using TRIOS 3 intra-oral scanner (3Shape, Copenhagen, Denmark). Color and spatial calibration were performed prior to scanning for each participant according to the manufacturer’s protocol. PLY model files were exported from the scanner for analysis using Geomagic Design X software (Artec, Senningerberg, Luxembourg). The 3D arrangement of the mandibular first molars was measured using 3 variables: CA, CI, and depth of the curve of Spee (DCOS).

-Maximum bite force measurement

Subjects were set to sit upright, keeping their heads straight, looking forward with their feet placed flat on the floor in a perpendicular orientation to the floor (9). MBF at first molars on the right and left were measured sequentially, with a 2-minute rest time between each measurement to avoid muscle fatigue (10,11). Three measurements were made on each side and the greatest value of three times was recorded as MBF on that side (9,11,12)

Three-dimensional arrangement of the first mandibular measurement 

-The digital measurement included 2 steps:

Step 1: Three-dimensional coordinates reorientation of 3D virtual models

**Figure 2:**
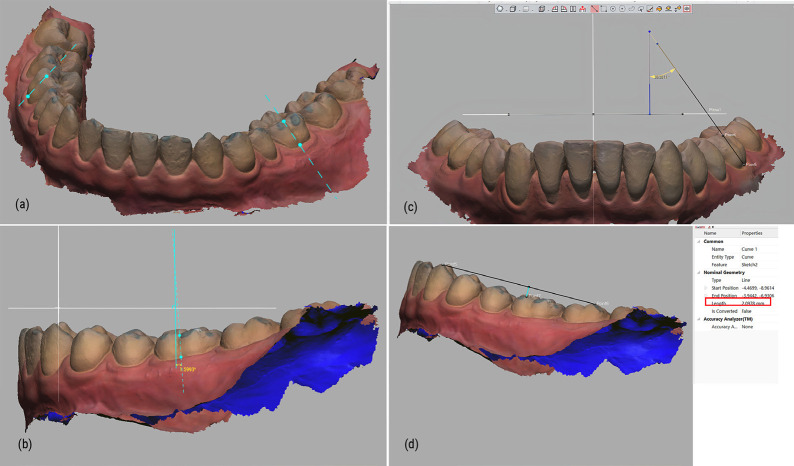
Three-dimensional coordinates reorientation of virtual models. Point A and point B were defined by the intersections of the palatal sulci and the gingival margin of the first maxillary permanent molars; The origin O was determined as the midpoint of the incisive papilla. The plane OAB served as the horizontal plane or Oxy plane. The sagittal (Oyz) and frontal (Oxz) planes were established perpendicular to the Oxy plane.

On each maxillary virtual model, we identified three dental landmarks: A, B and O. Utilizing these points, we established the Oxy, Oyz, and Oxz planes, culminating in the formation of the reoriented Oxyz coordinate system (13). A detailed depiction of this process can be found in Fig. 2.

Step 2: 3D arrangement of the first mandibular molars measurement

**Figure 3:**
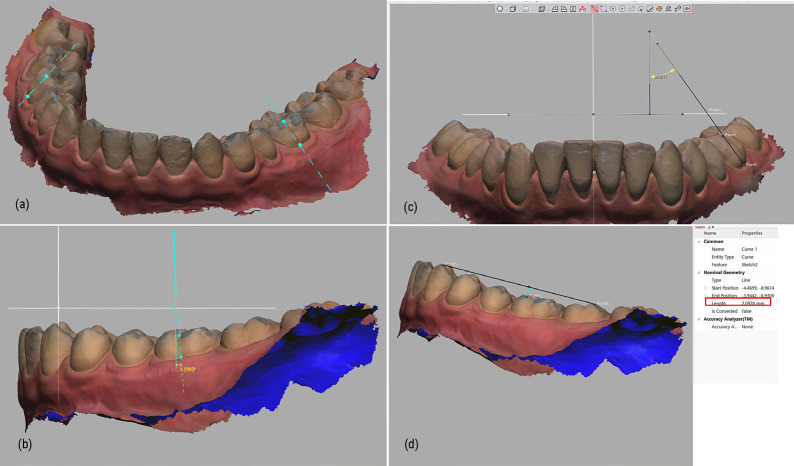
3D arrangement of the first mandibular molars measurement: (a) LACC of first mandibular molar definition; (b) Crown angulation (CA); (c) Crown inclination (CI); (d) Depth of Curve of Spee (DCOS).

First, the long axis of clinical crown (LACC) of each first mandibular molar was identified as a line parallel to the dominant vertical groove on the buccal surface (14) CA was the angle created by LACC and frontal plane (Oxz). CI was the angle between LACC and the sagital plane (Oyz) (15). DCOS was the projected distance from the mesiobuccal cusp tip of the first mandibular molar to the line joining the distobuccal cusp tip of the mandibular second molar and the tip of the canine on the sagittal plane (Fig. 3) (16,17)

To determine intra-examiner reliability, ten percent of total samples were randomly selected for repeated measurements after two weeks by the same observer.

-Statistical analysis

Statistical analysis was performed using the SPSS 20.0 (IBM, Armonk, NY, USA). A paired t-test was used to compare the means between sides (left and right) and a two-sample t-test was used to compare between genders (male and female). Pearson’s correlation coefficient was performed to evaluate the statistical relationships. The level of significance was set at 0.05.

## Results

-Description of maximum bite force, crown inclination, crown angulation, depth of curve of Spee

 Table 1 described the mean measurement values for all variables across genders and sides. No statistically significant differences were found between the sides. Regarding the left side, males’ mean MBF was found to be higher than females, although this disparity did not reach statistical significance. Conversely, considering either the right side or both sides, males’ mean MBF was statistically significantly greater than females (*P*<0.05).

Notably, there were no statistically significant differences in the mean CA, mean CI, or mean DCOS observed between sides or genders (*P*>0.05). Although both the mean CA and mean CI of males were greater than those of females, these differences were not statistically significant.

Intra-rater reliability of 3D arrangement measurements was analyzed by Cohen’s Kappa κ=0.712 (substantial agreement).

-Correlations between maximum bite force at first molars and crown inclination, crown angulation, depth of curve of Spee

**Figure 4:**
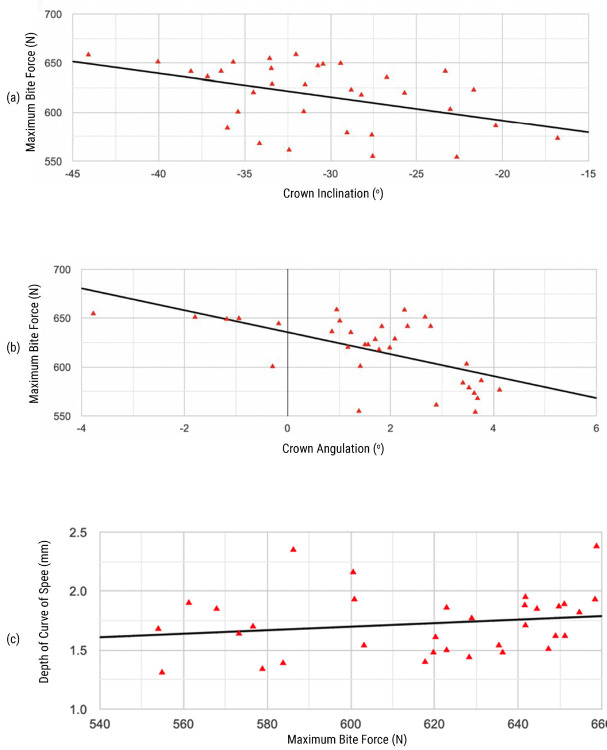
Correlations between the MBF at first mandibular molars and (a) crown angulation CA, (b) crown inclination (CI), (c) depth of curve of Spee (DCOS).

Fig. 4 illustrated the correlation between the MBF and 3D tooth arrangement. In both the female group and the total group, the correlation coefficient between the MBF and CA was statistically significant (*P*<0.05). Similarly, the correlation coefficient between MFB and CI was statistically significant in the female group and the combined gender group (*P*<0.05). However, no statistically significant relationship was found between MBF and DCOS.

## Discussion

Evaluating the integrity and function of the masticatory system is an important responsibility of dental practitioners. This requires a thorough examination and methodical evaluation of the masticatory system. Among them, an important contributor is bite force, because it is the result created by the coordination between different components of the masticatory. Furthermore, dental treatments often involve alterations in dentition arrangement, potentially impacting bite force. Hence, it is necessary to evaluate the relationship between bite force and tooth position, especially the key teeth like mandibular first molars. Our findings have revealed that changes in CA or CI of mandibular first molars can lead to variations of MBF in these regions, underscoring the significance of this relationship.

To explain the significant correlation coefficient between MBF and CI, CA, study showed that the CA and CI determine the occlusal plane and the occlusal contact areas, thereby affecting the MBF. These findings are consistent with previous studies suggesting that the arrangement of teeth on dental arches, the inclination of the crowns, or the relationship of the teeth between the two jaws affect the bite force (4). Earlier, Baragar and Osborn also suggested that if the occlusal surfaces of the posterior teeth are arranged harmoniously when the posterior teeth incline more forward, and the bite force is directed parallel to the tooth axis, the masticatory function on each molar is optimal (5).

Although the tilt of mandibular first molars in sagittal plane (Oyz) and frontal plane (Oxz) had affected MBF statistically, its position along the Oz in the sagittal plane which was evaluated in DCOS had no significant impact on MBF value in this current study. The correlation between MBF and DCOS was not significant, similar to the results of Fukei (7). However, in other studies, it was suggested that the arrangement of teeth according to the COS would be the most effective alignment for maintaining maximum tooth contact during mastication,16 and individuals with greater DCOS tend to exert greater force in functional movements (3). Moreover, bite force had a significant positive correlation with the radius of the occlusion curve, meaning people with greater bite force have smaller DCOS (18). In consistent with this finding, a study investigating the correlation between occlusal curves and chewing activity indicated that individuals with smaller DCOS would have faster chewing cycles as well as better grinding and mixing efficiency (7).

The mean MBF at first molars in our study is greater than MBF in Abu’s study (2010), Sasaki’s (1989); and smaller than some others such as Serra’s (2013) (12,19,20). This is because MBF varies depending on multiple factors such as tooth arrangement, age, gender, type of occlusion, dental history, facial type, and measurement protocol (1). Therefore, it is not easy to study the relationship of one single factor to MBF. And with this concern, we recommend setting suiTable inclusion criteria based on scientific evidence. For example, bite force may increase linearly from 6-18 years old due to physical growth, stay relatively stable after development, and decrease gradually after the age of 25 in females and 45 in males (1). COS also changes with age (21). Hence, subjects in the current study are selected between 18-25 years old, the period at which the DCOS is relatively sTable, bite force reaches its peak, and the dentition has not been affected by physiological wear (21). Regarding gender, the mean MBF of males was found significantly greater than females, in accordance with previous researches.20,22,23 This was explained by the differences in body weight and hormones between genders (1).

In addition, it is necessary to take into account the status of the teeth in MBF investigation, because various researches indicated a significantly higher MBF on sound teeth in comparison with restored or endodontic-treated teeth (1,24). Another finding showed that the occlusal force is highest in healthy teeth, reduced to 80% in fixed restorations, 35% in partial removable restorations, and only 11% in full removable restorations (25). These changes are explained by adaptation of the periodontal ligament after tooth preparation, or by soft tissue discomfort when wearing dentures (1). Finally, measuring tools and protocols had an important influence on the result. For instance, the design of the bite platform in this study had a thickness of 11 mm, consistent with the conclusion that the MBF could be achieved when the intermaxillary gap at molar regions was from 9-20 mm (1,26). Besides, the bite platform was laminated with a plastic layer and a foam layer on each side, in order to reduce the fear of injury for participants, which was shown to generate greater bite force compared to a hard surface (20).

For accurate anatomical variable measurements, such as DCOS, CA and CI, it’s imperative to standardize and align all the models into the same reference planes, ensuring reduced technical errors and enhanced repeatability. Specifically, in measuring DCOS, aligning all models into a common sagittal plane view, as outlined by Spee, is crucial (16). The 3D measurement method offers an advantage in this regard, allowing for easy standardization of landmarks and view adjustments, marking an improvement over cameras and plaster models. However, reference planes have varied across studies. While cranial planes, like palatal plane for the maxillary incisor, or the mandibular plane for the mandibular incisor, are valuable for tooth inclination assessments, they necessitate radiographic measurements. The occlusal plane is another potential reference but is deemed unstable, potentially causing misalignment, especially in longitudinal or post-treatment studies due to potential changes from aging or treatment (13).

In this study, we standardized all virtual models using Ferrario’s three-dimensional frame method, a technique previously applied in plaster model studies and 2D photo analyses for crown angulation and crown inclination assessments, and for evaluation of the curve of Wilson (27,28). This method is favored for its stability, ease of definition, and its ability to obviate the need for supplementary data collection, like radiographs (13). Digital analysis further mitigates errors from silicone impressions, plaster model creation, and conventional calipers measurements, especially with extensive datasets. Additionally, the wear of gypsum models during transportation and storage can skew results, making digital storage a space-efficient, damage-resistance, and time-saving alternative.

In this study, while moderate to strong correlation was observed between MBF and the orientation of first mandibular molars, the study’s population was limited to individuals with Angle’s class I occlusion. Given the influence of occlusion on MBF and the arrangement of dental arches, future research should explore this relationship across various occlusion types to ensure broader population representation. Even though we controlled the multi-factors affect on MBF, in order to improve from our suggested inclusion criteria, we recommend considering facial type in the recruitment of participants. It is reported that individuals with short-face patterns tend to have the highest MBF, followed by the average-face type and long-face type; because the thickness of the masseters in the short-face group is larger than that of the other types (12).

## Conclusions

In conclusion, the association between MBF and tooth position warrants further investigation with broader participant recruitment. We propose that our digital protocol holds promise for dental morphology research and could be performed automatically using advanced technologies such as artificial intelligence for large-scale population analysis, potentially yielding more significant insights.

## Figures and Tables

**Table 1:** Description of maximum bite force (MBF), crown angulation (CA), crown inclination (CI) and the depth of curves of Spee (DCOS) measured at the mandibular first molars and their correlation with gender.

Variable		Males	Females	Total	P ^a^
n		16	17	33	
Age		22.01 ± 1.28	22.05 ± 1.77	22.03 ± 1.15	0.62
MBF (N)	Right	640.48 ± 18.99	600.07 ± 38.06	619.66 ± 36.25	<0.001***
	Left	622.71 ± 36.73	606.74 ± 39.83	614.47 ± 38.61	0.12
	Both sides	631.62 ± 22.54	603.44 ± 36.07	617.11 ± 33.07	0.006**
CA (°)	Right	2.25 ± 1.90	2.58 ± 1.50	2.21 ± 1.70	0.49
	Left	1.58 ± 1.75	2.26 ± 1.59	2.09 ± 1.68	0.07
	Both sides	1.91 ± 1.60	2.42 ± 1.20	2.15 ± 1.41	0.15
CI (°)	Right	-29.88 ± 6.18	-29.43 ± 7.75	-29.65 ± 6.93	0.43
	Left	-30.44 ± 6.79	-30.40 ± 6.79	-30.42 ± 6.68	0.49
	Both sides	-30.16 ± 5.66	-29.91 ± 6.76	-30.03 ± 6.16	0.45
DCOS (mm)	Right	1.75 ± 0.24	1.70 ± 0.35	1.73 ± 0.30	0.31
	Left	1.75 ± 0.30	1.70 ± 0.35	1.72 ± 0.32	0.34
	Both sides	1.75 ± 0.21	1.70 ± 0.35	1.72 ± 0.27	0.30

a: two sample t-tests in comparison between males and females Significance levels: *: *P*<0.05 **: *P*<0.01 ***: *P*<0.001

## Data Availability

The datasets used and/or analyzed during the current study are available from the corresponding author.
